# Preparation of efficient photothermal materials from waste coffee grounds for solar evaporation and water purification

**DOI:** 10.1038/s41598-020-69778-2

**Published:** 2020-07-29

**Authors:** Chih-Feng Wang, Chih-Lin Wu, Shiao-Wei Kuo, Wei-Song Hung, Kuo-Jung Lee, Hsieh-Chih Tsai, Chi-Jung Chang, Juin-Yih Lai

**Affiliations:** 1 Advanced Membrane Materials Research Center, Graduate Institute of Applied Science and Technology, National Taiwan University of Science and Technology, Taipei, 106 Taiwan; 2 R&D Centre for Membrane Technology, Chung Yuan University, Taoyuan, 320 Taiwan; 3 Department of Materials Science and Engineering, I-Shou University, Kaohsiung, 840 Taiwan; 4 Department of Materials and Optoelectronic Science, National Sun Yat-Sen University, Kaohsiung, 804 Taiwan; 5 Department of Chemical Engineering, Feng Chia University, Taichung, 407 Taiwan

**Keywords:** Renewable energy, Solar energy, Materials chemistry

## Abstract

Effective water use is currently a critical global challenge needed to prevent water shortages and has attracted significant research attention. The realization of solar-driven water evaporation by using effective converters has attracted considerable attention in recent years owing to its potential for seawater desalination and wastewater treatment. Consequently, this paper proposes a simple two-step method to prepare low-cost and self-floating photothermal converters from waste coffee grounds. First, the coffee grounds were carbonized at 1,000 °C to develop broadband absorption, and the carbonized coffee grounds were modified using hydrophobic silane to enhance the water-floatation property of the grounds. The prepared hydrophobic carbonized coffee grounds exhibited good performance for desalination and water purification under sunlight irradiation. The self-floatation ability, low cost, well solar evaporation performance, and easy preparation contribute to the promising potential of using hydrophobic carbonized coffee grounds infuture steps toward eco-friendly desalination procedures.

## Introduction

Water scarcity is one of the most critical global challenges currently faced by humanity, and has thus attracted significant attention and academic research^1,2^. Despite over 67% Earth’s surface being covered by water bodies, only a small proportion is available for human consumption and use^3^. Previous studies have investigated membrane-based traditional desalination technologies that can produce potable water from seawater, including membrane distillation (MD), electro-dialysis reversal (EDR), and semi-permeability in reverse osmosis (RO).However, these approaches are not economically viable in remote areas due to their high energy consumption^4^.

Sunlight is a renewable energy source and can be utilized in a solution addressing clean water scarcity with negligible environmental impact such as solar-driven water evaporation. However, the process has a low light-to-vapor conversion efficiency that hinders the application of this technique, largely due to the poor optical absorption of water and the acute losses associated with the process. In response, numerous materials have been developed for efficiently absorbing light and converting it to heat to generate water vapor^5–16^. Nanostructured metals have been implemented for solar absorption and as conversion materials, and have been found to be highly efficient due to their localized surface plasmon resonance effects. Yang et al. prepared a bilayer Janus film by incorporating gold nanorods in an interconnected carbon nanotube porous film that exhibited high efficiency of photothermal conversion^17^. Liu et al. synthesized a plasmonic-active filter paper using iron to heat and attach gold nanoparticles to filter paper, which was successfully used for solar-to-vapor generation with high evaporation efficiency^18^. More recently, carbon-based materials have been prepared and applied in the solar-driven water evaporation process^19–23^. However, many of these materials either involve expensive composites or follow complicated fabrication protocols that limit their up-scaling and application. The challenge remains to develop a new material for solar evaporation with low cost, self-floatation ability, and easy production.

Coffee ranks as the second most valuable product in the world after petroleum and its derivatives, and the coffee industry generates a large amount of refuse^24^. The International Coffee Organization reported that approximately 9 million metric tons of coffee are created globally each year. However, it should be noted that waste coffee grounds are composed of cellulose-based materials that are thought to be suitable precursors for fabricating carbon materials^25^.

In this work, aiming for a sustainable economic process, we report a facile two-step method to fabricate hydrophobic carbonized coffee grounds from waste coffee grounds as an efficient solar receiver for solar water vapor generation. First, we performed the carbonization process to enhance the pore structure and the carbon content of the coffee grounds. Subsequently, hydrophobic silane was coated on the surface of the carbonized coffee grounds to introduce hydrophobic properties. It was noted that the as-prepared hydrophobic carbonized coffee grounds could self-float on the water surface without any assistance and achieved a fairly high efficiency for water vapor generation under an irradiation of1 sun (1,000 W/m^2^). Finally, the hydrophobic carbonized coffee grounds were also used for saline water desalination, and it was noted that the ions in the condensed fresh water could be significantly reduced. The expected simple preparation, self-floatation ability, and high evaporation efficiency could mean that the hydrophobic carbonized coffee grounds could satisfy the requirements of practical applications.

## Results

**Scheme 1:**
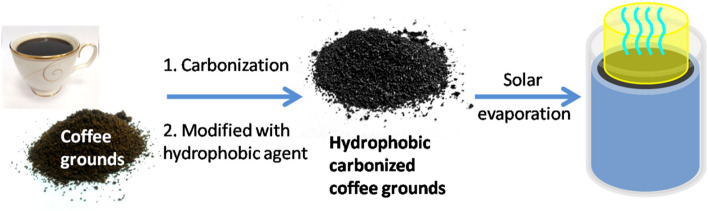
Schematic of the process flow for the preparation of the HCCG.

**Figure 1:**
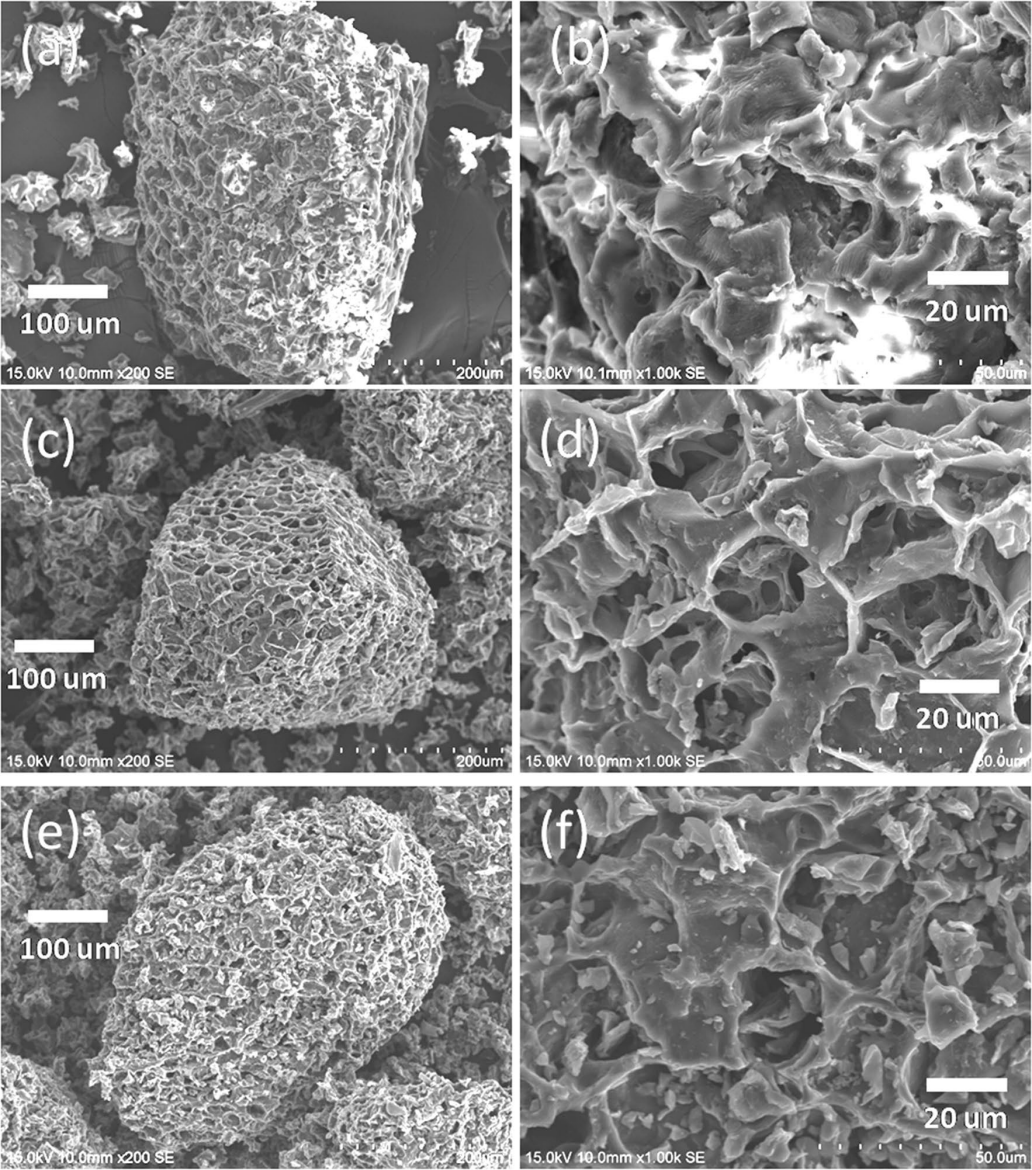
SEM images of (**a**,**b**) CG, (**c**,**d**) CCG, and (**e**,**f**) HCCG.

**Figure 2:**
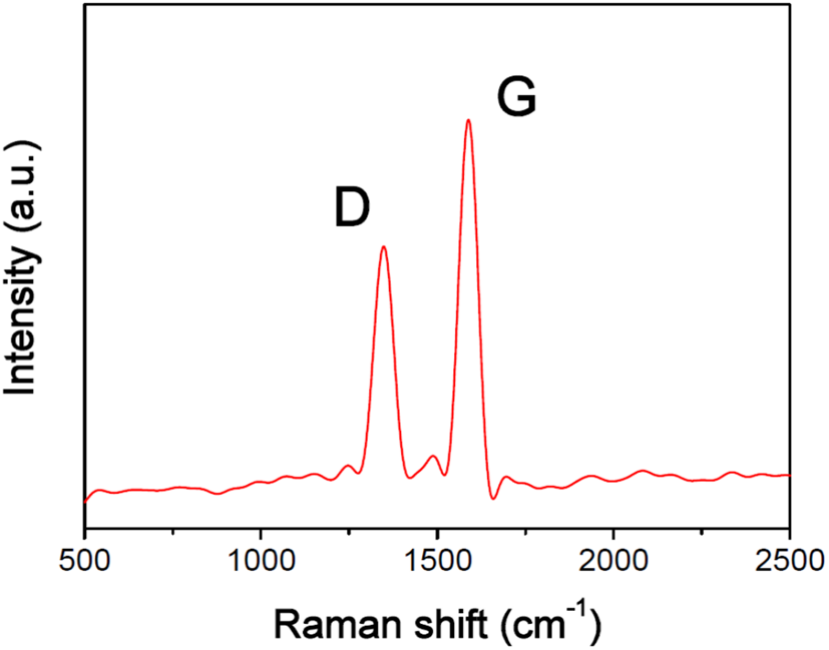
Raman spectrum of the CCGs.

Herein, we reported a simple method to develop particle-based materials for solar evaporation through waste coffee grounds (Scheme 1). First, the waste coffee grounds were carbonized at 1,000 °C to prepare the carbonized coffee grounds. Then, the carbonized coffee grounds were modified using dodecyltriethoxysilane to enhance the hydrophobicity and self-floatation ability. Figure 1 shows the SEM images of the coffee grounds (CG), carbonized coffee grounds (CCG), and hydrophobic carbonized coffee grounds (HCCG). These SEM micrographs show that the coffee grounds possessed a porous structure (Fig. 1a,b). Figure 1c,d indicate that the porous structures and surface roughness of the CG were enhanced through carbonization. After the modification using the hydrophobic agent (dodecyltriethoxysilane), the morphology of the CCG did not exhibit significant changes (Fig. 1e,f).The Raman spectrum of the CCG shows the characteristic bands of the G and D bands at ∼1588 cm^−1^ and ∼1,348 cm^−1^, respectively (Fig. 2), which can be attributed to the sp^2^ hybridized graphitic carbon atoms and defected carbon atoms, respectively.

**Figure 3:**
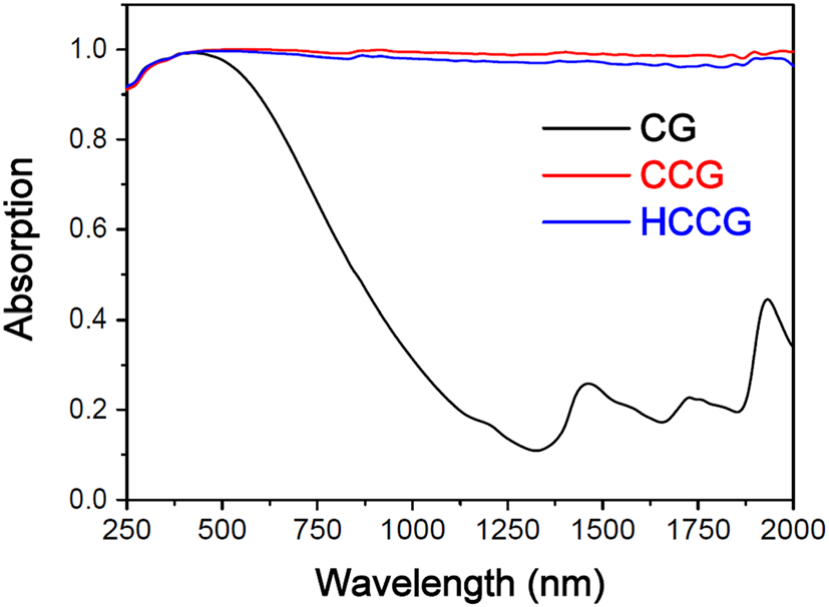
Absorption spectra of the CG, CCG, and HCCG at wavelengths ranging from 250−2000 nm.

EDX analysis was used to elucidate the elemental compositions of CG, CCG, and HCCG. The EDX analyses show that the major elements present in the CG and CCG were carbon (C) and oxygen (O). For pristine coffee grounds, the carbon content was 63.40%. After carbonization, the carbon content was considerably enhanced to 91.05%.After the surface modification, the carbon content slightly decreased to 88.79%, and the signal of Si was noted on the hydrophobic carbonized coffee grounds. This finding indicated that the carbonized coffee grounds were coated with the hydrophobic agent (dodecyltriethoxysilane) successfully through the surface modification. Figure 3 displays the UV–Vis-NIR absorption spectra (250–2000 nm) of the CG, CCG, and HCCG samples. We could note that the carbonization process significantly increased the broadband light absorption and was beneficial in realizing a high solar thermal conversion. The surface modification of the CCG induced only a slight loss of light absorption.

**Figure 4:**
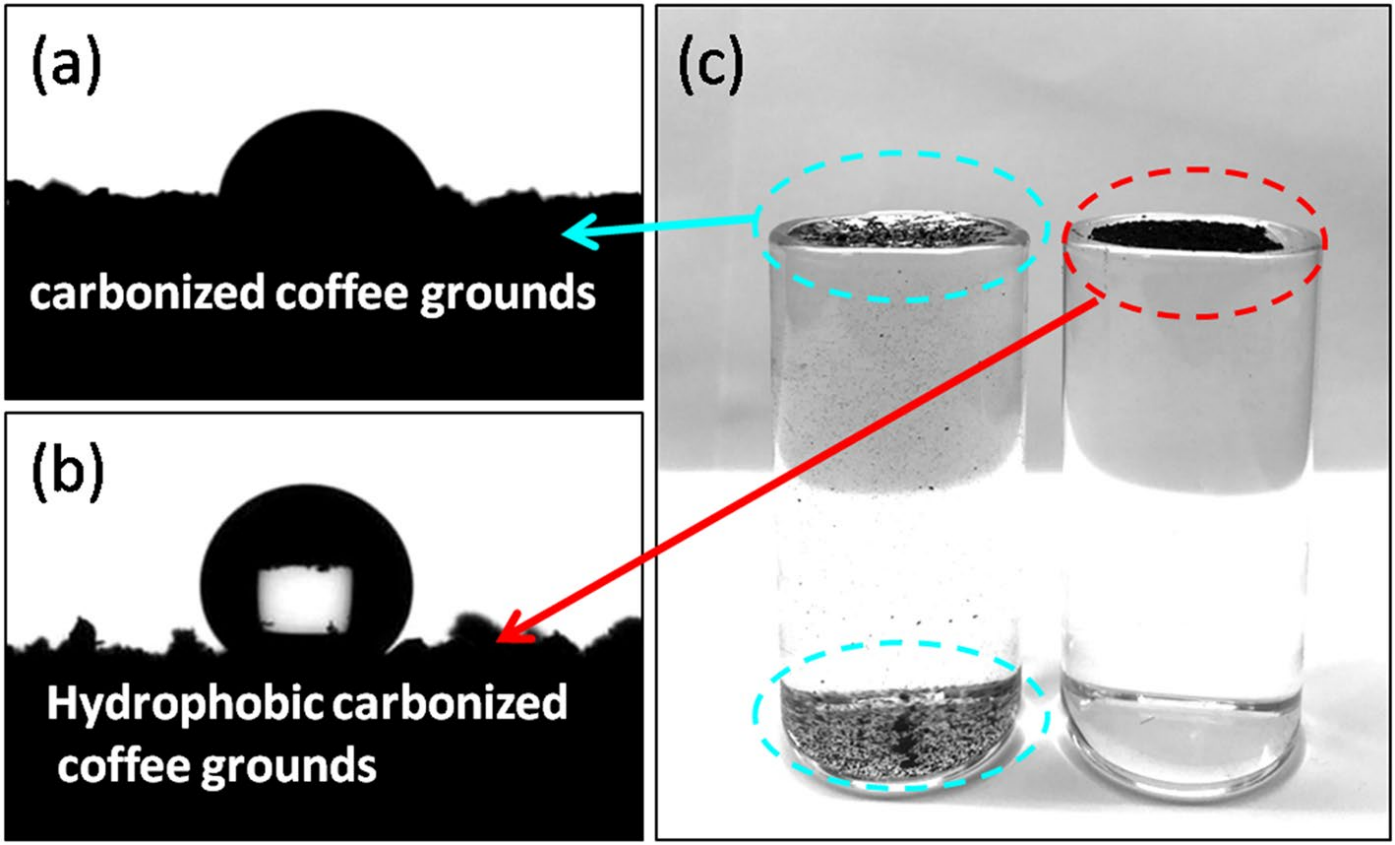
Profile of water drops on the (**a**) CCG and (**b**) HCCG. (**c**) Image of the water-floatation test.

The materials used in solar evaporation systems must have self-floatation ability. It is difficult for the solar light to reach the materials effectively when the particles are immersed below the surface of the water, as the high heat capacity of water prevents heat conversion on the materials. In our study, the coffee grounds were imparted the water-flotation ability by modifying them using a hydrophobic agent. To investigate the wetting behaviors of CCG and HCCG toward water, we employed a contact angle goniometer to measure the contact profile of the water droplets. Figure 3 shows the water contact angle of the CCG and HCCG. The CCG exhibited hydrophilicity with a contact angle of approximately68° (Fig. 4a). In the water floatation test, the CCG initially floated on water surface; however, a large amount of CCG was noted to be immersed in water after being in contact with water for 10 min because of the hydrophilicity of these grounds (Fig. 4c). After contacting with water for 30 min, all CCGs were sunk at the bottom of the test container. The water contact angle of the HCCG was approximately 144° (Fig. 4b). This finding indicates that the surface modification of CCG considerably increased their hydrophobicity. Furthermore, the surface modification of CCG also considerably enhanced their self-floatation ability.

**Figure 5:**
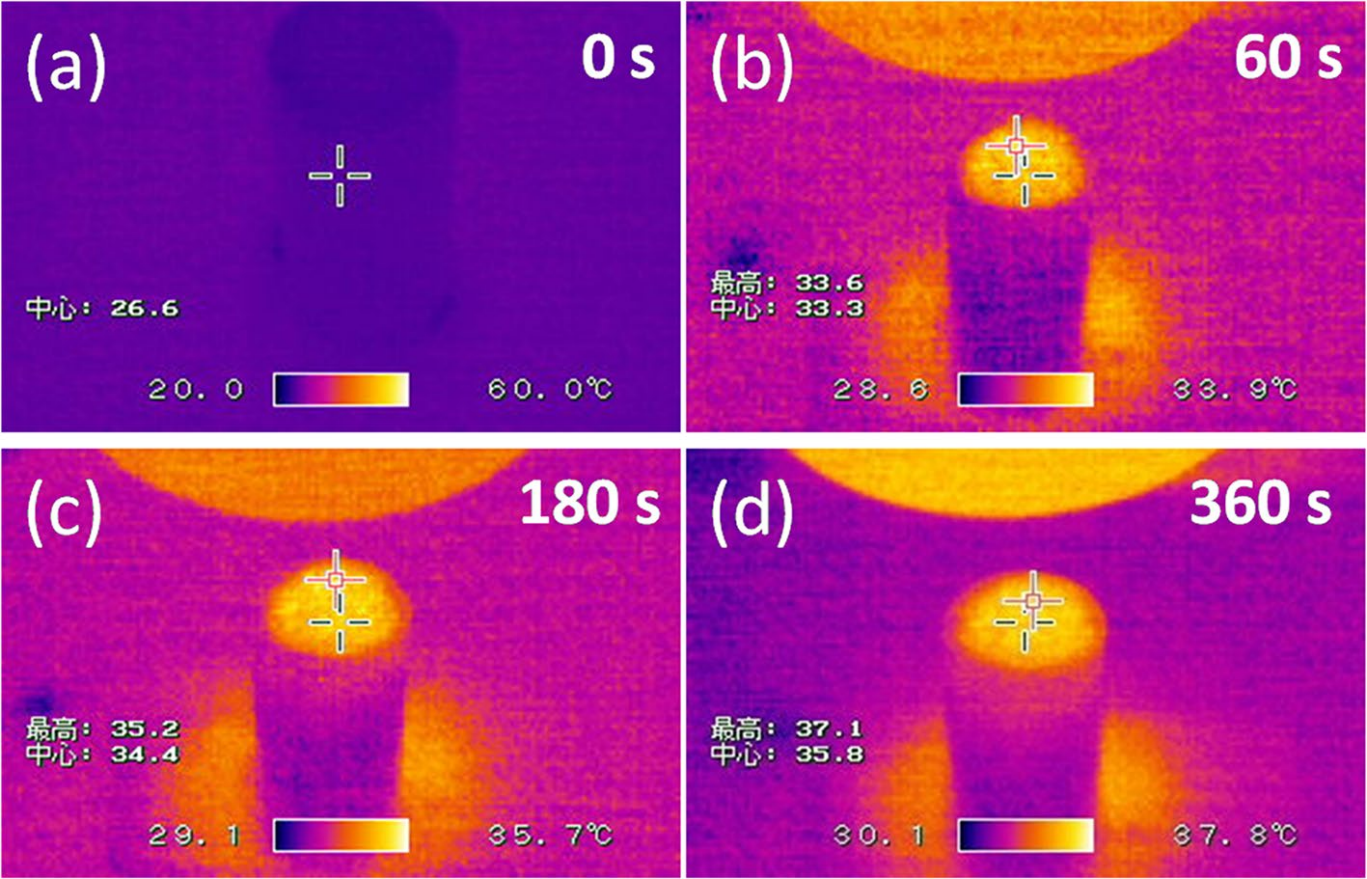
Infrared thermal images of the water sample covered with HCCGs after 1 sun irradiation for (**a**) 0, (**b**) 60, (**c**) 180, and (**d**) 360 s.

The photothermal properties of the hydrophobic carbonized coffee grounds were studiedusing an IR camera to detect the temperature of the particles under 1sunirradiation (Fig. 5). This demonstrated the solar heating occurring at the air–water interface in the solar evaporation procedure. The surface temperature of the self-floating HCCG was *ca.*26 °C before light illumination (Fig. 5a).With 1 sun irradiation, the interfacial water temperature with the HCCG increased instantly (Fig. 5b,c). The interfacial water temperature increased with longer irradiation times and a steady-state temperature up to 37 °C was achieved after irradiation of 360 s and beyond (Fig. 5d).This indicates that the HCCG will efficiently absorb solar irradiation, convert it to heat energy and increase the temperature of the water at the water–air interface. This promotion of thermal conversion will lead to more efficient solar evaporation.

**Figure 6:**
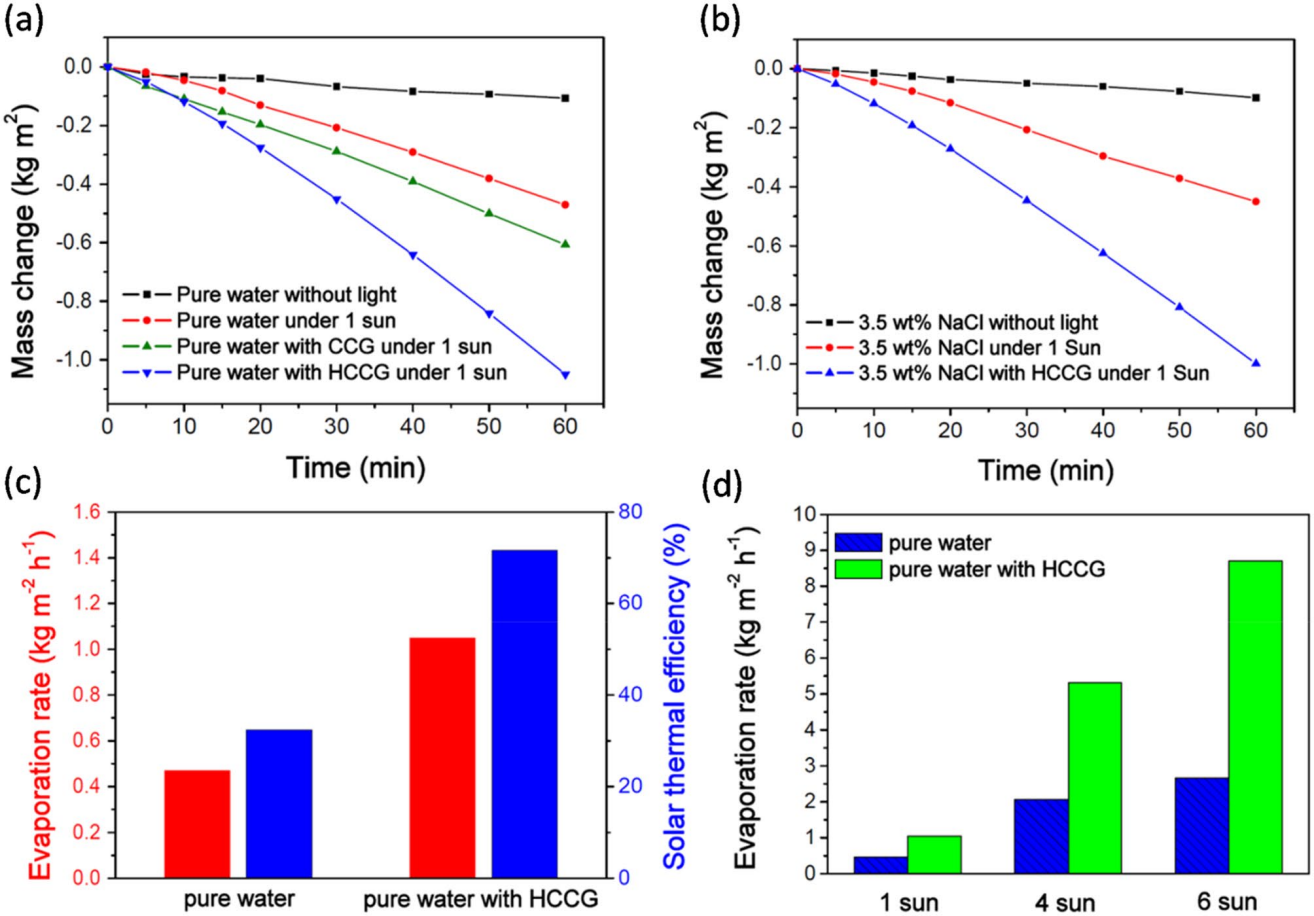
Change in mass of the (**a**) pure water and (**b**) 3.5 wt %NaClover time in a dark field and under 1 sun irradiation. (**c**) Solar thermal efficiencies (blue, right-hand side axis) and corresponding evaporation rates under 1 sun irradiation (red, left-hand side axis) with the HCCG. (**d**) Evaporation rates of pure water with and without HCCG under different illumination intensities.

In actual use, the water evaporation performance is evaluated using the actual apparent mass changes. These changes were simply estimated for the HCCG by placing the sample on the water surface and recording the evaporation rates of the systemthrough measuring the weight loss of the water with the progression of time (Fig. 6). Figure 6a shows the mass change with the irradiation time for a pure water evaporator, a CCG-based evaporator (in this system, all CCGs were sunk in the bottom of the test container), and a HCCG-based evaporator under 1 sun irradiation as well as in the dark. The mass loss rate of natural water without light irradiation was 0.107 kg/m^2^h. Under 1sun irradiation, the mass loss rate of water for the HCCG/water system was 1.05 kg/m^2^h, which is approximately 2.23 times that of pure water (0.471 kg/m^2^h) and 1.73 times that of CCG/water system (0.607 kg/m^2^h). It confirmed that the self-floatation ability is important for the materials used in solar evaporation systems. We also performed the solar evaporation test with 3.5 wt% NaCl (Fig. 6b). Under1 sun irradiation, the evaporation rate of water for HCCG/3.5 wt% NaCl system was0.998 kg/m^2^h.This finding indicated that the hydrophobic carbonized coffee grounds could demonstrate satisfactory thermal conversion performance in both pure water and 3.5 wt% NaCl systems.

In this work, the energy conversion efficiency (η) was calculated using

1 $$\eta =\dot{m}{h}_{LV}/I$$

where $\dot{m}$ is the evaporation rate of water under steady-state conditions (kg/m^2^h), ${h}_{LV}$ is the total enthalpy of liquid–vapor phase change, and* I* is the power density of solar irradiation (1,000 W/m^2^). ${h}_{LV}$ contains two parts, namely, sensible and latent heat enthalpies, which can be calculated as

2 $${h}_{LV}= \Delta {h}_{vap}+ {C}_{p}\Delta T$$

where $\Delta {h}_{vap}$ is the latent heat of vaporization of water under standard atmospheric pressure, ${C}_{p}$ is the specific heat capacity of water (4.2 kJ/kg K), and ΔT is the change of water temperature. Furthermore,$\Delta {h}_{vap}$ is dependent on the temperature and can be determined from a specialized database^22,26,27^. The solar thermal efficiency for water under 1 sun irradiation was 32.4%, which increased to 71.7% in the case of HCCG (Fig. 6c). This demonstrated that the HCCG can perform highly efficient solar evaporation under 1 sun irradiation. The evaporation rates of the HCCG under solar irradiation values of one, four, and six sun were 1.05, 5.32, and 8.71 kg/m^2^h, respectively, which represented values 2.2–3.3 times compared to those of pure water (Fig. 6d).Compared with many other kinds of photothermal materials, our HCCG possesses the advantages of low cost, good solar evaporation performance, and easy preparation (Table 1).

**Table 1:** Comparison of various carbon-based photothermal materials used for solar evaporation.

Materials	Solar intensity (sun)	Evaporation rate (kg/m^2^h)	Refs.
Carbonized tissue membrane	1	0.86	^19^
Carbon-nanotube-based floating solar still	1	0.88	^21^
Surface carbonized wood	1	1.08	^29^
Flame-treated wood	1	1.05	^30^
Carbon nanotube-modified flexible wood membrane	1	0.95	^31^
Hydrophobic carbonized coffee grounds	1	1.05	This work

**Figure 7:**
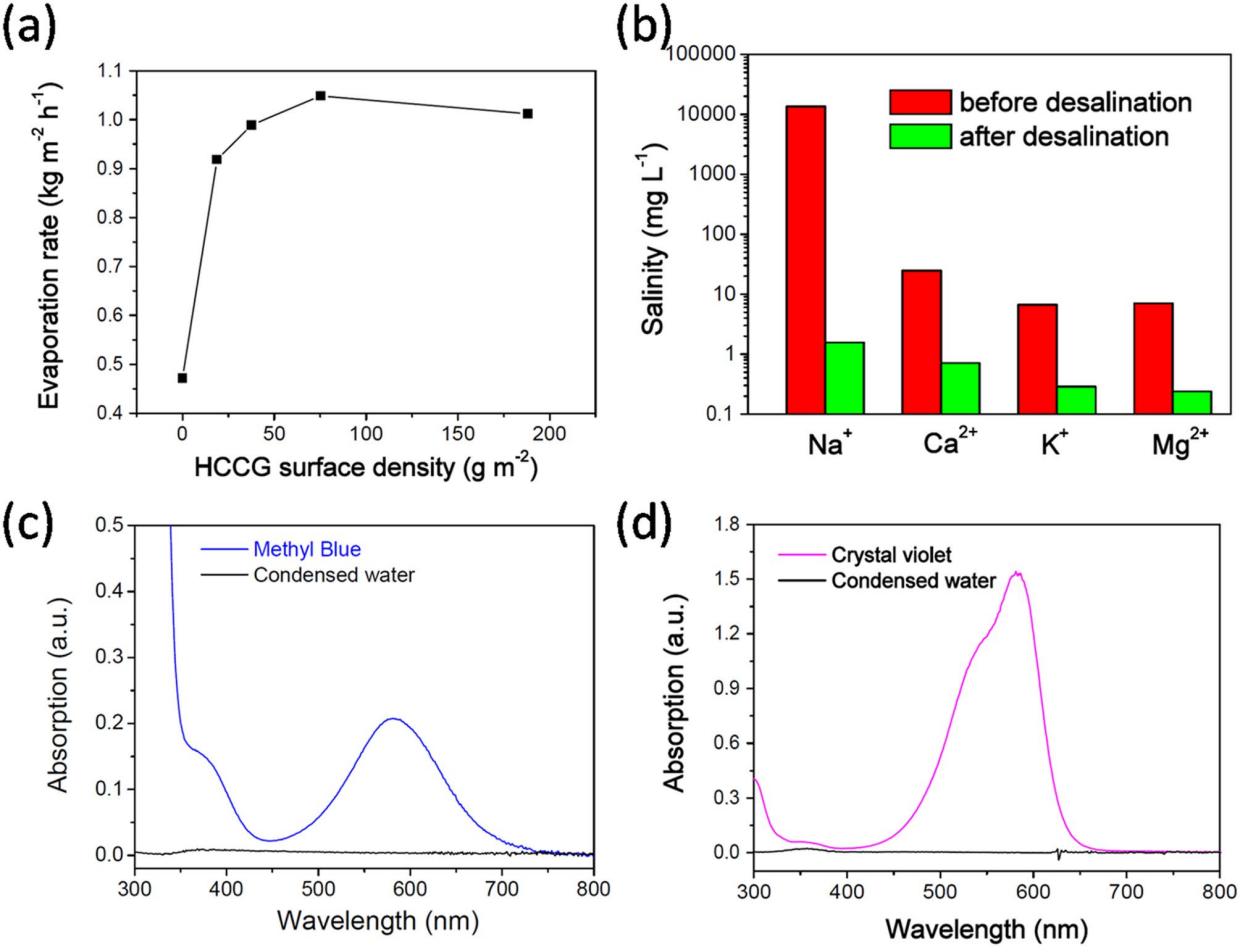
(**a**) Plot of evaporation rate as a function of the surface density of the HCCG under 1 sun irradiation. Water purification performance pertaining to HCCG under solar irradiation. (**b**) Concentrations of four types of primary ions in the simulated seawater(Na^+^, Ca^2+^, K^+^, and Mg^2+^) before and after desalination. (**c**,**d**) The sewages (methyl blue or crystal violet aqueous solutions) are condensed using a solar-thermal energy converter

Various quantities of HCCG were dispersed onto the water surface to measure the solar thermal efficiency, whereby the surface area was based on the container used (2.66 cm^2^). The water mass change was measured as time goes on. Figure 7a shows the evaporation rates of water for different surface densities of HCCG under 1 sun irradiation for 1 h. During solar evaporation, the evaporation rates increased with the increase in the surface density of the HCCG until they plateaued at 75.2 g/m^2^ and decreased thereafter. This phenomenon was a result of the limitations of both heat and mass transfer. When the thickness of the absorption layer was excessively thick, heat could not be properly transferred to the HCCG layer–water interface at which the water vapor was generated. Thus, there is an optimal surface density of the light absorber at the surface of the water for effective solar evaporation. A similar observation was made regarding previously reported modified diatoms used for solar evaporation^28^.

**Scheme 2:**
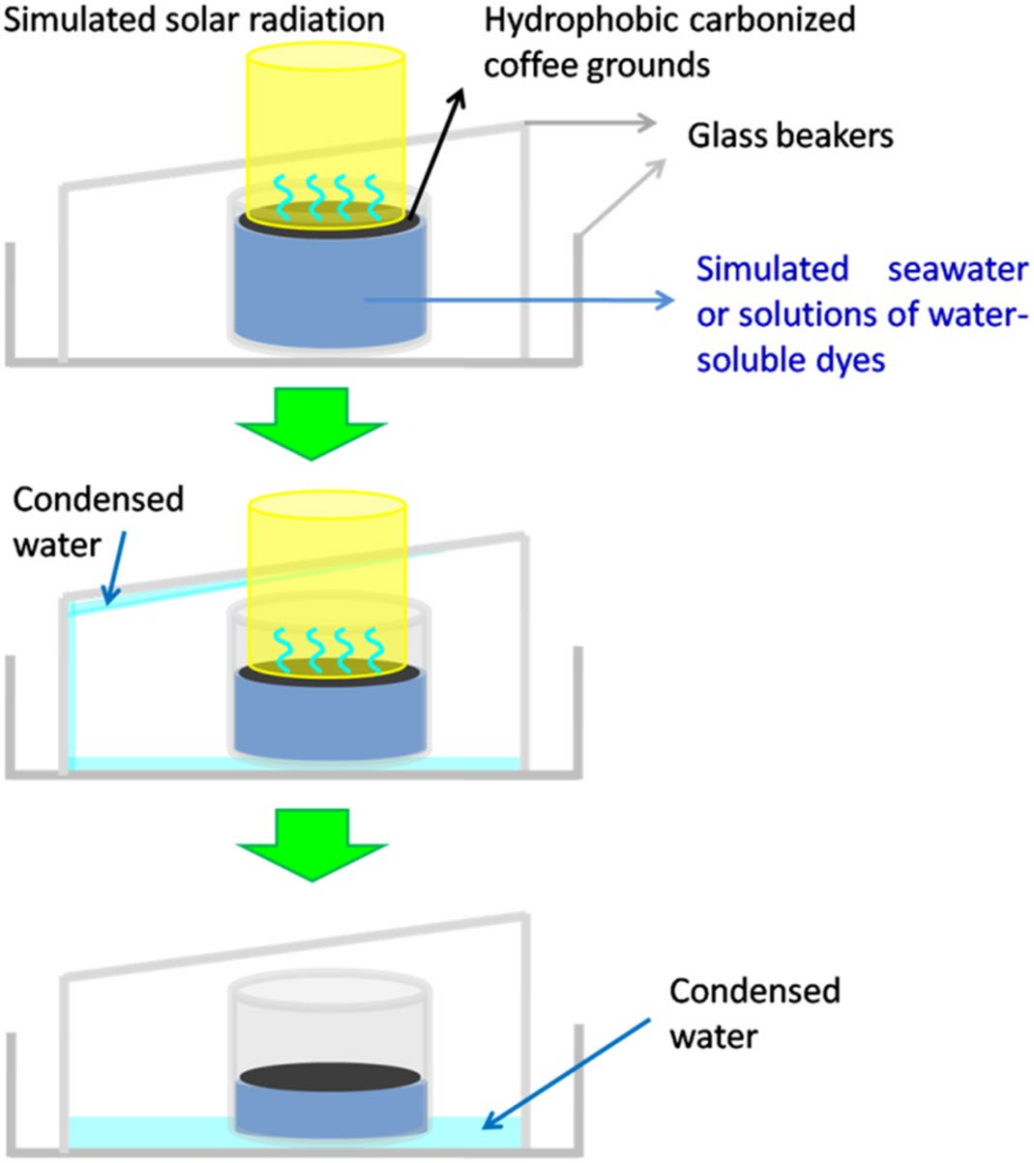
Schematic of the device for the desalination and the sewage treatment.

**Figure 8:**
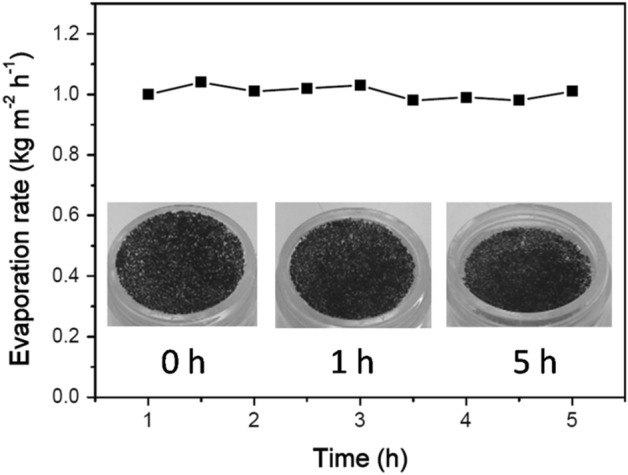
The evaporation rates of 3.5 wt% NaCl as a function of irradiation time under1 sun irradiation with the HCCG.

A simple device was constructed to conduct solar desalination and water purification, as shown in Scheme 2.The desalination performance of the HCCG for 3.5 wt% NaCl was evaluated based on the ion concentrations of Na^+^, Ca^2+^,K^+^,and Mg^2+^after desalination detected using ICP-OES (Fig. 7b). After desalination, the concentrations of all four ions were reduced to a level considerably below the safe-drinking-water limits provided by the World Health Organization (WHO). Therefore, the hydrophobic carbonized coffee grounds could provide an effective technique for solar desalination, which can not only produce clean water from 3.5 wt % NaCl, but also exhibit satisfactory performance for the purification of sewage (methyl blue or crystal violet aqueous solutions with the concentration of 50 ppm). The quality of the generated clean water was confirmed using the UV–vis spectra, which indicated that the contaminants were negligible, as indicated by the near-zero optical absorbance (Fig. 7c,d). This clearly demonstrated the potential of the hydrophobic carbonized coffee grounds for application in solar seawater desalination and sewage purification. The durability is an important characteristic for the photothermal conversion materials for real-environment application. We used a long-term stability test to evaluate the durability of the HCCG for desalination. In this test, a HCCG-based evaporator was continuously operated with 3.5 wt% NaCl under 1 sun over 5 h. For the HCCG-based evaporator, there was no evident decay in the evaporation rates during the continuous illumination (Fig. 8). Furthermore, we did not observe any salt on the surface of the HCCG after 5 h’s desalination. The hydrophobicity of HCCG can avoid the crystallization of salts on the surface^32^, it can enable stable solar desalination.

## Discussion

We reported upon a facile two-step method to prepare low-cost and self-floating photothermal converters from waste CG. The as-prepared HCCG possessed a great broadband light absorption from 250 to 2000 nm. Under1 sun irradiation, the evaporation rate of water for the HCCG/water system was1.05 kg/m^2^h, which corresponded to a conversion efficiency of 71.7%, thereby demonstrating well performance for desalination and water purification. After the desalination of the 3.5 wt % NaCl, the ion concentrations of Na^+^, Ca^2+^, K^+^, and Mg^2+^ reduced sharply at a level considerably less than the safe-drinking-water values provided by the WHO. The presented experimental results may provide a novel opportunity for the fabrication of eco-friendly desalination devices from biomass-based photothermal conversion materials.

## Methods

### Fabrication of hydrophobic carbonized coffee grounds

The waste coffee grounds obtained from beverage manufacturers were washed with acetone and n-hexane sequentially and then dried at 90 °C for 2 h. The washed coffee grounds were placed in a small quartz tube, which was then placed at the center of a tubular furnace. Under a nitrogen atmosphere, the furnace was heated to 1000 ºC for 1 h with a heating rate of 3 ºC min-1. After the furnace cooled down to room temperature, the carbonized coffee grounds were extracted. Dodecyltriethoxysilane (Gelest, Inc.) was chosen as the hydrophobic agent. Surface modification of the carbonized coffee grounds was performed by treating the sample ultrasonically in an ethanolic solution of dodecyltriethoxysilane (1 wt%) for 1 h and subsequently baking it at 120 °C for 1 h.

### Characterization

The microstructures and energy-dispersive X-ray spectroscopy (EDX) analysis of the coffee grounds, carbonized coffee grounds, and hydrophobic carbonized coffee grounds were characterized using a scanning electron microscope (SEM, HITACHI S-4700). The static contact angles of the droplets (5 μL) were measured using an FDSA Magic Droplet-100 contact angle goniometer. An IR camera (Thermo Shot F30, Nippon Avionics Co., Ltd.) was employed to measure the temperature changes. The concentrations of Na^+^, Ca^2+^, K^+^, and Mg^2+^ in the desalinated water and saline water were measured using plasma atomic emission spectrometry (ICP-OES, Agilent 720, Agilent Technologies Inc.). The UV–vis adsorption spectra were measured using a UV/VIS/NIR spectrometer (V-770, JASCO).

### Water vapor generation

Solar simulators (XES-151S, San-Ei Electric, and Sirius-300P, Zolix) were used as light sources for the solar evaporation generation experiments. The intensity of light was measured using the X1-1 optometer (Gigahertz-Optik GmbH). The hydrophobic carbonized coffee grounds, in quantities of 20 mg, were allowed to float on vessels containing 22 g of distilled water. The changes of mass were recorded using an electronic analytical scale, and the values were recorded in real-time by a computer. For all the evaporation experiments, the humidity and temperature and were maintained between 56−63% and 25−28 °C, respectively.
